# Dietary fiber and growth, iron status and bowel function in children 0–5 years old: a systematic review

**DOI:** 10.29219/fnr.v67.9011

**Published:** 2023-03-27

**Authors:** Jutta Dierkes, Bright I. Nwaru, Alfons Ramel, Erik Kristoffer Arnesen, Birna Thorisdottir, Christel Lamberg-Allardt, Ulrike Spielau, Fredrik Söderlund, Linnea Bärebring, Agneta Åkesson

**Affiliations:** ^1^Centre for Nutrition, Department of Clinical Medicine, University of Bergen, Bergen, Norway; ^2^Department of Laboratory Medicine and Pathology, Haukeland University Hospital, Bergen, Norway; ^3^Krefting Research Centre, Institute of Medicine, University of Gothenburg, Gothenburg, Sweden; ^4^Faculty of Food Science and Nutrition, University of Iceland, Reykjavik, Iceland; ^5^Department of Nutrition, Institute of Basic Medical Sciences, University of Oslo, Oslo, Norway; ^6^Faculty of Sociology, Anthropology and Folkloristics & Health Science Institute, University of Iceland, Reykjavik, Iceland; ^7^Department of Food and Nutrition, University of Helsinki, Helsinki, Finland; ^8^Department of Internal Medicine and Clinical Nutrition, Institute of Medicine, Sahlgrenska Academy, University of Gothenburg, Gothenburg, Sweden; ^9^Unit of Cardiovascular and Nutritional Epidemiology, Institute of Environmental Medicine, Karolinska Institutet, Stockholm, Sweden

**Keywords:** dietary fiber, children, iron, growth

## Abstract

**Background:**

While dietary fiber intake is low in many children, the current trend to plant-based diets is associated with higher fiber intake in children raised on these diets. As older reports indicate that diets providing high fiber intake in children 0–5 years may affect growth, iron status and bowel function, we summarized the available evidence in this systematic review.

**Objective:**

To identify, critically appraise, and synthesize evidence on the effect of high fiber intake on growth, iron and bowel function in children 0–5 years, with relevance to the Nordic and Baltic countries.

**Methods:**

Following a pre-registered protocol, we searched MEDLINE, EMBASE, Cochrane Central of Controlled Trials, and Scopus for clinical trials and prospective cohort studies published until November 2021. Two reviewers independently screened retrieved literature, extracted relevant data, and performed risk of bias assessment. Outcomes were growth, iron metabolism and bowel function in children 0–5 years. We narratively described findings from studies that met inclusion criteria.

**Results:**

From 5,644 identified records, five articles met the inclusion criteria. Two RCTs had an overall moderate risk of bias, while the three observational studies had serious risk. Overall, we found no robust association between high intake of dietary fiber and growth. In the RCTs, higher intake of fiber had a positive effect on bowel movements and constipation. No studies on fiber intake and iron status were identified.

The certainty of the overall evidence was inconclusive for growth and bowel function, while no assessment was made for iron status.

**Conclusion:**

We found no clear association between high intake of dietary fiber and growth or bowel function in young children living in affluent countries, albeit with only a limited number of studies. There is a lack of studies investigating health effects of high fiber intake in small children.

## Popular scientific summary

- High dietary fiber in small children may be associated with lower risk for constipation, but also with reduced growth and iron deficiency
- We searched the literature for studies of high fiber intake in children living under affluent conditions, but identified a very limited no. of studies on bowel movements and growth, and non on iron deficiency
- Supplementation of dietary fiber led to higher stool frequency and softer stools in small children. The association of high fiber intake in young children with growth remains unclear

Emerging evidence shows that high fiber intake has beneficial effects on bowel function, cardiometabolic risk factors and cardiometabolic risk in adults, while the benefits are less clear in children (1). In their scientific opinion on carbohydrates and dietary fiber, EFSA concluded that the optimal amount of dietary fiber in children is unknown but considered an intake of 2 g per MJ to be adequate for normal laxation in children from the age of 1 year (1). Dietary fiber recommendation for children, if in operation, varies between countries, but are often in the range of 10 to 19 g/day for 1–3 year olds (2). Reported dietary fiber intake in children (<10 years) are usually in the range of 10–15 g per day and in many reports lower than the dietary recommendations (1, 2). Indeed, there is only limited knowledge on dietary fiber intake in pre-school children, as most reports cover older children and adolescents. However, it is suggested that dietary habits in childhood, once they have developed, will persist from adolescence to adulthood (3).

While low fiber intake is related to constipation (4), high fiber intake may increase food volume and thus compromise energy intake. Furthermore, bioavailability of divalent cations is reduced. This may result in reduced growth in small children and may also affect iron status. As most reports on high fiber intake are from low-and-middle income countries with diets often inadequate in other nutrients, it is unclear whether this would apply to otherwise well-nourished children in affluent countries. A high dietary fiber intake in children is usually due to a plant-based diet (5, 6) or for example, other dietary patterns like macrobiotic diets (7–9), which could be deficient in other nutrients, potentially explaining any association with impaired growth and development (10). These concerns have been summarized in a narrative review from 1995 (11) which however concluded that even doubling the current intake of dietary fiber will give more benefit than harm. Indeed, there are few studies on higher fiber intakes in children not suffering from undernutrition, as also stated in recent systematic review focusing on high fiber intake in children and intermediate markers of cardiometabolic risk and other health issues (12).

The role of high dietary fiber intake in young children and their putative effect on growth and development, iron status and bowel function was identified as one of the prioritized topics among the systematic reviews commissioned by NNR Committee for the update of the 2012 Nordic Nutrition Recommendations (13). A priori published criteria (14, 15) included that a new systematic review was warranted when important new scientific data have been published, no recent qualified systematic review on the topic exits and the topic was of relevance to the Nordic or Baltic countries.

Following a scoping review, the NNR Committee concluded that with an increasing prevalence of plant-based diets and high fiber intake, the role of high fiber intake on growth and development, iron status and bowel function in small children should be investigated in a systematic way.

Hence, the aim of this systematic review was to identify, critically appraise and synthesize evidence from studies on the role of high fiber intake on growth and development, iron status and bowel function in small children living in affluent countries.

## Methods

The systematic review process followed the guidelines developed for the NNR 2022 (16). The systematic review process also followed the recommendation of the Preferred Reporting Items for Systematic Reviews and Meta-analyses (PRISMA) (17, 18). First, the NNR 2022 Committee developed, using an iterative process with the authors, a focused systematic review question, which included definition of the study population, intervention/exposure, control, outcome, timeframe, study design and settings (PI/ECOTSS) (Table 1). A protocol was pre-registered online on PROSPERO (CRD42021288211).

**Table 1:** The inclusion criteria for the literature search, the population/participants, intervention/exposure, control, outcome, timeframe, study design and settings (PI/ECOTSSPICOTTS)

Population	Intervention or exposure	Comparators	Outcomes	Timing	Setting	Study design
Children (6 months to 5 years)	Dietary fiber, total and sub-groups (these subgroups could be soluble and in-soluble fiber; the fractions determined by chemical analyses; or based on the source: e.g. grain, pulses, vegetables or fruits)	High vs low. dose-response Only focusing on consequences of high intake (per quartile or increase per g fiber intake)	Bowel function (constipation / diarrhoea) Growth focusing on BMI/BMI *z*-score, weight for age, Length for age. Iron status	bowel function: Short time/few days of follow-up, depending of study design and outcome Growth: minimum 6 months follow- up, in children <1 year: 3 months Iron status: minimum 3 months	Relevant for the general population in the Nordic and Baltic countries Age-range 6 months – 5 years of the children	Prospective cohort studies, interventions, RCTs

BMI = body mass index

RCT = randomized controlled trial

The study was funded by the Nordic Council of Ministers and governmental food and health authorities of Norway, Finland, Sweden, Denmark, and Iceland.

### Eligibility criteria

We included studies in healthy children from 6 months up to 5 years living in settings comparable to the Nordic and Baltic countries (Table 1). We considered dietary fiber intake as defined in the articles without applying a standard definition of fiber.

The study designs of interest were randomized (RCT) or non-randomized intervention trials, and prospective cohort studies. Required duration of studies was dependent on the study design and the outcome of interest: observational studies on bowel function must have at least 4 weeks duration while the required duration for interventions was 2 weeks. Studies on growth and development must have a minimum follow up of 6 months and at least 3 months in infants, and studies on iron status must have a duration of at least 3 months. For intervention studies, studies were included if the intervention was compared to usual diet, in the absence of dietary advice or nutrient supplementation, or placebo/other comparators used. In cohort studies, comparison was made to lower intake (e.g. quantiles). Studies only including only treatment of constipation were excluded.

### Information sources and search strategy

A comprehensive search strategy of MEDLINE, EMBASE, Cochrane Central of Controlled Trials, and Scopus was made by research librarians at Karolinska Institutet University Library, and peer reviewed by the University of Oslo Library of Medicine and Science, up to November 2021. The search strategy (Supplementary file 1) was developed in collaboration with the authors, led by JD. There were no exclusions by publication date or language. The reference list of included studies was also screened to identify potentially eligible studies.

### Selection and data collection process

All literature retrieved from the database searches were exported to Endnote for de-duplicating, followed by export of the remaining papers to the web-tool Rayyan (https://rayyan.qcri.org) for literature screening. The literature screening was performed by two members (AR and EA) of the team, working independently. Literature screening was first piloted with approximately 10% of the obtained titles and abstracts before full literature screening on the remaining 90% of the papers. If at least one of the reviewers voted for inclusion, the paper was included in the full text screening. Potentially eligible papers were retrieved and read in full text by the two reviewers. Discrepancies between assessors were resolved by discussion or by a third reviewer (AÅ).

Data extraction was made by two reviewers independently (AR and JD), using pre-specified Excel forms.

Any discrepancies in the data extraction were resolved by discussion. The data extraction form included: the full reference, study design, information dietary intake, interventions and controls, assessment of outcomes, follow-up, drop-out, and confounders on recruitment.

### Study risk of bias assessment

Two reviewers (BT and JD) independently evaluated the risk of bias in all included studies. Any discrepancies were resolved by discussion. For observational studies, assessment of risk of biased was based on the ‘Risk of Bias for Nutrition Observational Studies’ (RoB-NObS) tool (developed by the USDA’s Nutrition Evidence Systematic Review [NESR]) alone (19). The domains assessed with RoB-NObS are, as with ROBINS-I, confounding, selection of participants, classification of interventions/exposures, deviations from intended interventions/exposures, missing data, measurement of outcomes, and selection of the reported result. The risk of bias in each individual study was classified as low risk, moderate, serious or critical both at each domain of bias assessment, and overall. The details for considerations for grading of each domain of the study and overall grading are provided in the RoB-NObS document, it should be noted that a study is judged to be at high risk of bias overall if one of its domains has a high risk of bias grading. For RCTs, the Cochrane’s risk of bias 2.0 Tool was used (20).

### Synthesis methods

Included studies were synthesized in a narrative review including the characteristics and context of the studies, their strengths and limitations, heterogeneity (in study characteristics and results) and relevance. Main outcomes for each outcome are listed in table form. Following the recommendations of the Agency for Healthcare Research and Quality (AHRQ) and the Cochrane Handbook, our priori criteria to performing meta-analysis stipulated that more than three independent RCTs or five cohort studies must be available on each specific question for a meta-analysis to be undertaken (21, 22). In addition to not meeting these conditions given fewer studies, high heterogeneity between the included studies precluded any meta-analysis.

### Certainty assessment

Strength of evidence was categorized according to the World Cancer Research Fund’s grading: ‘Convincing’, ‘Probable’, ‘Limited – suggestive’, ‘Limited – no conclusion’, ‘Substantial effects unlikely’ (14) The quality (risk of bias), quantity, consistency, and precision in the body of evidence were used for categorizing the strength of the evidence. A *convincing* body of evidence was established as strong enough to support a causal relationship or lack of a relationship in which several conditions are met, including evidence coming from more than one study type. A *probable* body of evidence was supported when strong enough to support a probable causal relationship and there was evidence from at least two independent cohort studies, no unexplained heterogeneity between or within study types, good-quality studies to confidentially exclude possible random or systematic errors, and evidence for biological plausibility. A *limited – suggestive* was supported when there was evidence from at least two independent cohort studies, a consistent direction of effect, and evidence for biological plausibility. A *limited – no conclusion* evidence was established if the evidence is so limited that no firm conclusion could be made. Any evidence strong enough to support a convincing absence of a causal relationship was considered *substantial effects unlikely*.

## Results

### Study selection and search results

**Fig. 1:**
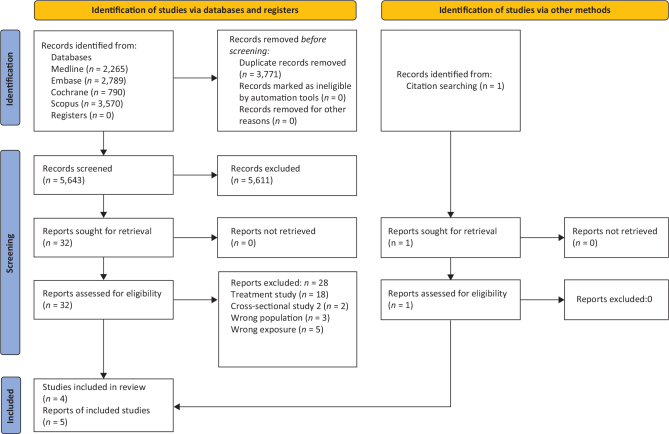
PRISMA flow diagram of database searches and study screening and selection.

A total of 5,643 records were retrieved from the database searches after de-duplication, of which 5,611 were excluded after title and/or abstract screening. Of the 32 full-text papers evaluated, four met the criteria to be included in the review (originating from two RCTs (23, 24) and two papers from one cohort (25, 26)). One additional cohort study was identified by hand-search (27). Figure 1 gives the flowchart for the literature screening. Reasons for excluding each of the remaining 28 studies after full-text screening are included in Supplementary file 2.

While both RCTs and one observational study addressed bowel function, and both RCT and two observational studies addressed growth, we did not identify eligible studies on high fiber intake and iron status. The observational cohort studies reported data from 2,420 participants from the Generation R study and 543 participants from the STRIP study (Table 2). The RCTs included 19 and 56 participants, respectively.

**Table 2:** Included cohort studies

Name of cohort and country	Author and year	Population	Sample size	Exposure and dietary assessment	Outcomes	Follow up time	Total RoB
Generation R (Netherlands)	Kiefte-de Jong 2013	Healthy children (born from 04/2002 to 01/2006)	*N* = 2,420 (no. analyzed)	Dietary fiber intake at 14 months (FFQ)	Constipation / Stool pattern / Bowel function at 14, 24, 26, and 48 months	24, 36 and 48 months of age	Serious
Generation R (Netherlands)	Van Gijssel 2016	Healthy children (born from 04/2002 to 01/2006)	*N* = 2,032 (no. analyzed)	Dietary fiber intake at 12.9 months (FFQ)	BMI Body fat % At 6 years	Age 6 years	Serious
STRIP study*	Routtinen 2010	Healthy children	*N* = 543 (no. analyzed)	Dietary fiber intake from dietary records	Growth from 8 months to 2 years Weight from 13 months to 9 years	9 years	Serious

* The STRIP study was started at a randomized study, but the current analysis is as a longitudinal cohort study. ROB, risk of bias.

FFQ = food frequency questionnaire

### Narrative review

#### RCTs: study designs and outcomes

We included two RCTs, both conducted in the US. One study investigated 19 healthy children aged 2–5 years in a 4-weeks cross over study where the children got a commercial raisin bran supplement containing 5 g fiber for 2 weeks and 10 g fiber for the following 2 weeks. The control group received a spread enriched with plant sterols (24). The main outcome was blood lipids, but weight and height as well as stool production and weight were measured at baseline and end of each study period and reported.

The other study was a parallel RCT including 56 healthy infants aged 4–11 months who in a double-blind fashion received infant cereals containing oligofructosaccharides (0.75 g/portion (25 g cereals)) or control cereal for 4 weeks. Main outcomes were stool frequency, color and consistency, flatulence, anthropometric measurements at baseline and at the end of the study (23) (Table 4).

**Table 3:** Summary of findings from cohort studies

Author, year	Age at outcome	Outcomes reported	Findings	Effect size
Kiefte-de Jong, 2013	24, 36 and 48 months	Constipation: 8–13% between 24 and 48 months	Baseline fiber intake in children with subsequent constipation: 17 ± 9 g/day Baseline fiber intake in children without subsequent constipation: 18 ± 9 g/day	*P* > 0.05
Van Gijssel, 2016	6 years	% Body fat BMI not reported	Association of energy-adj. dietary fiber intake (per 1 g/day increase) with % body fat	-0.003 (-0.0015 – 0.010) (95% confidence interval)
Routtinen, 2010	2 years 9 years	Length and weight at 2 years Weight development from 13 months to 9 years	Weight gain increased by 34 g per 1 g increase in fiber Low fiber (<10th perc.) 10.3 to 30.3 kg Average fiber (10th to 90th perc) 10.2 to 30.8 kg High fiber (>90th perc) 10.3 to 31.0 kg	*P* = 0.032 n.s.

**Table 4:** Characteristics of randomized clinical trials

Reference	Design	Country	Population	Outcomes	Intervention	Control	Sample size	Duration	RoB
Williams 1999	Cross-over study	USA	Healthy children, 2–5 years	Stool frequency per week Stool weight on specified days Weight and height	Kellog raisin bran: 2 weeks providing 5 g/day dietary fiber 2 weeks providing 10 g/day dietary fiber	Plant-Stenol ester containing spread	*N* = 19 (analyzed)	Each period 4 weeks, 2 weeks wash out	Moderate
Moore 2003	RCT	USA	Healthy infants 4–11 months, mean age 31.8 ± 9.0 weeks (control), 34.7 ± 8.9 weeks (intervention)	Stool frequency, color and consistency Flatus, vomiting, colic, Weight, length	At least one meal/day with Cereal porridge (25 g/day) with fructo-oligosaccharides (0.75 g/25 g)	At least one meal cereal porridge (25 g) without FOS, but added maltodextrin	*N* = 56 (27 + 29)	28 days	Moderate

ROB, risk of bias; FOS, fructo-oligosaccharides.

Additional dietary fiber from a commercial raisin bran increased both the stool frequency and the stool weight but had no effect on body weight (24). Addition of fructo-oligosaccharides (FOS) FOS increased the stool frequency and led to less likely hard stools and more likely soft, but not watery stools. Stool pH was not significantly different. Development of weight and height was not different between the groups (23) (Table 5).

**Table 5:** Results from randomized clinical trials

Author and year	Outcomes reported	Fiber intervention	Control	*P*	Adverse events
Williams 1999	Stool frequency: Baseline 7.25 ± 2.94 /week Stool weight (on specified days): Baseline (ounces) 1.80 ± 0.75 Body weight	4 weeks 8.11 ± 2.60 /week 3.05 ± 1.33 ounces +0.30 ± 0.72 pounds	4 weeks 6.77 ± 1.97 / week 1.92 ± 0.81 ounces +0.56 ± 0.71 pounds	0.014 0.001 0.31	23 gastrointestinal events in the spread phase and the bran phase, respectively, no adverse events
Moore 2003	Stool frequency /d Stool consistency Stool pH Weight change Length change	1.99 ± 62 / day Less likely heard, more likely soft, but not watery 6.1 ± 0.77 +0.56 ± 0.23 kg +20 ± 13 mm	1.58 ± 0.66 /day 6.4 ± 0.94 0.54 ± 0.24 +16 ± 12 mm	0.02 0.01 n.s. n.s. n.s.	No difference in non-serious adverse events between intervention and control group, no serious events

#### Observational studies

We included three publications based on two observational cohorts – the Finnish STRIP study (27) and two publications from the Generation R study from the Netherlands (25, 26).

Dietary intake in the Generation R study was assessed at a median age of 12.9 months (25) or at mean age of 14 months (26) by a validated food frequency questionnaire (FFQ). While Kiefte-de Jong studied association of fiber intake with parental reports of constipation at 24, 26 and 48 months of age in 2,420 children, the focus of van Gijssel was on body composition and other cardiometabolic risk factors at age 6 years, measured in 1,988 children.

Ruottinen reported associations of fiber intake with weight from the Finnish STRIP study (Special Turku Coronary Risk factor intervention project in children) (27). Although originally a randomized intervention study, the analysis on dietary fiber was independent of the intervention group. Intervention was dietary counselling on a healthy diet in regular intervals and the control group was usual care on diet. Dietary fiber intake of children was reported from 3 or 4-day dietary records.

In the Generation R study, dietary fiber intake at 14 months was not related to constipation at 24, 36 or 48 months of age. However, the authors reported an association of Western dietary patterns with constipation.

Dietary fiber intake at 12.9 months was associated with a favorable cardiometabolic score (of which % body fat was one component) at age 6 years. The association with % body fat was not significant.

In the STRIP study, dietary fiber intake was positively and significantly associated with weight development from 8 months to 2 years, but weight of children with low (<10th perc), average (10th to 90th perc) and high (>90th perc.) fiber intake was not different at age 9 years. Results are summarized in Table 3.

#### Risk of bias in included studies

**Fig. 2:**
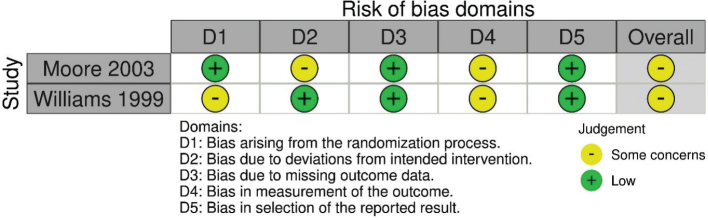
Risk of bias assessment in RCTs using RobVis.

Of the five included studies, the observational studies received an overall serious risk of bias grading, while the RCTs received a moderate risk of bias grading. The lowest gradings were given for risk of bias due to selection of participants, and for risk of bias due to departures from intended exposures (Fig. 2 and Table 6).

**Table 6:** Risk of bias grading in observational cohort studies following the RoB-Nobs tool

Study	D1	D2	D3	D4	D5	D6	D7	overall
Kiefte- de Jong 2013	Moderate	Serious	Low	Serious	Moderate	Low	Low	Serious
Van Gijssel 2016	Moderate	Serious	Low	Serious	Moderate	Low	Low	Serious
Routtinen 2010	Serious	Serious	Low	Serious	Moderate	Low	Moderate	Serious

ROB, risk of bias.

D1 Bias due to confounding.

D2 Bias in selection the participants into the study.

D3 Bias in classification of exposures.

D4 Bias due to departures from intended exposures.

D5 Bias due to missing data.

D6 Bias in measurement of outcomes.

D7 Bias in selection of reported result.

#### Certainty in the evidence

*Growth *On the basis of two short term RCT (which were underpowered to assess growth) and two observational studies that did not show an association, we considered the certainty of this evidence as *limited – no conclusion*. We would like to mention that the fiber intakes even in the upper end percentiles in the observational studies was not specifically high.

*Iron status *Given the absence of any eligible study, it is impossible to judge the evidence.

*Bowel function *Of the three studies included, two RCT showed a significant result towards higher stool frequency and softer consistence in healthy children. The observational study showed no association. On the basis of a limited number of studies and contradictory findings, we considered the certainty of this evidence as *limited – no conclusion*.

## Discussion

### Summary of key findings

We identified very few studies that fulfilled our eligibility criteria. Overall, we found no robust association between high intake of fiber and growth in healthy infants and preschool children. Increased dietary fiber increased stool production in the RCTs, but higher fiber intake was not associated with constipation in the observational study. We did not identify studies on fiber intake and iron status.

### Comparison to previous studies

Previous studies were more concerned about low dietary fiber intake than high fiber intake.

In a narrative review, Williams concluded already in 1995 that doubling the dietary fiber intake would probably do more benefit than harm (11). However, representative data on fiber intake in infants and small children is limited. From NHANES, average dietary fiber intake in in children in the US aged 2–5 years has been estimated to be 11 g in 2–5-year-old boys and girls (28). Alexy et al. reported that energy-adjusted dietary fiber intake in German infants and children was highest during the second half of the first year (average and SD intake at 9 months: 2.97 ± 0.87 g/MJ and at 1 year 3.06 ± 0.90 g/MJ) and declined when children had their diet adopted to family habits (29). Many studies report low habitual fiber intake in preschool children (30).

Fiber intake in children in the Nordic countries has been evaluated in Norway in 2019 in children 12 months of age (31), in 2007 in children of 2 years of age (32) and in Sweden in 2003 in children 4 years old (33). Average fiber intake in 12 months olds was 17 ± 6 g/day, which was higher than in the previous investigation from 2009 (13 ± 5 g/day). The 90th percentile of fiber intake at 12 months was 26 g/day (32). In 2007, average dietary fiber intake of 2-year-old children in Norway was 17 ± 6 g/day (2.4 ± 0.6 g/MJ) (32). Taken the average fiber intake in 12-months old children in Norway, data are very comparable to those from the Generation R study that measured dietary intake at approximately the same age (26). Swedish data from 2003 showed that average fiber intake in 4 year olds was 11 g/day, with a 90th percentile of 16 g/day (33).

### Bowel function

Constipation is a common problem among infants and small children, and it is estimated that about 7–30% of children suffer from constipation (34) and there is evidence for an association of low fiber intake and risk of constipation (35).

Increasing fiber intake is the first option for treatment of constipation in children, even though there are studies that do not report an improvement of constipation at higher fiber intakes (28). Taylor reported on an inverse association of fiber intake and hard stools in 30–42-months old children from the ALSPAC cohort, but they did not investigate in particular high dietary fiber intake (30). In the ALSPAC cohort, average dietary fiber was low with 8.8 ± 2.9 g/day non-starch polysaccharides. The role of high fiber intake and constipation or bowel function has obviously not been investigated.

### Growth

Plant-based diets have been associated with higher fiber intakes in 5–10-year-old children (6). In this cross-sectional study, children on vegan diets had lower *z*-scores of height, BMI and fat mass than omnivore children, and lower markers of iron status. However, even though differences in fiber intake were obvious between groups, there are other dietary components that differ between a vegan and omnivore diet, leaving the role of high fiber intake unclear. It has to be mentioned that there was no study identified that specifically investigated high fiber intake and growth or iron status in small children following omnivorous diets, making conclusions on the effect of high fiber intake within such diets impossible. Given the current trend to plant-based diets also in children, studies are urgently needed to investigate the health effect of such diets in small children.

The association of fiber with obesity risk was beyond the scope of this systematic review. Even though usually, a high-fiber diet is regarded as associated with lower risk of obesity, there are studies that show that higher fiber intake is associated with higher energy intake (27), which should be further investigated.

### Interpretation and implications of findings

The importance of dietary fiber intake in children is based on their effect of bowel function and long-term health effects. Further, fiber is a constituent in foods that are recommended to be consumed – whole grain, legumes, nuts, and fruits and vegetables. All these foods also provide other nutrients and phytochemicals that are regarded as beneficial.

Recommendations for dietary fiber intake in children are given in a number of countries (2), either given as amount per day or as amount per energy intake. The different units also make comparisons of studies more difficult. It also turns out that the basis for these recommendations is either age plus 5 g/day fiber intake, as suggested by Williams (11), or in absolute numbers extrapolated from recommended fiber intake per energy intake in adults (28), resulting in either 2 g/MJ (1) or 14g/1,000 kcal (3.5 g/MJ) (36) or absolute numbers (1–3 years of age: 19 g/day, 4–8 years of age: 25 g/day). It is important to notice that these different recommendations do not align and may be reason for confusion (28). Thus, the scientific basis for dietary fiber in children is limited and more studies should investigate both the amount and the type of dietary fibers that are associated with gut health and other outcomes in children. Further, it has to be mentioned that according to these definitions, neither the average fiber intake nor the intake in children with the highest intake was particularly high. In the intervention studies, added fiber was 5–10 g/day (24), or 0.75 g/portion cereals. Average fiber intake in the Generation R study was either 18 ± 9 g/day (25) (at the mean age of 14 ± 2 months), or 15.0 ± 4.4 g/day (at the median age of 12.9 months) (26) and in the STRIP study, the average dietary fiber did not exceed 2.10 g/MJ between 13 months and 9 years of age. Children consuming >90th percentile had a consumption of 2.4 ± 0.7 g/day at 13 months and 2.6 ± 0.6 g/day at 5 years.

Thus, there is a lack of knowledge on the effects of higher fiber intakes on growth and iron status in children following either plant-based or omnivore diets.

### Strengths and limitations

In this systematic review, established processes for undertaking robust systematic reviews were followed, as established a priori by the NNR 2022 Committee. According to prespecified guidelines, a detailed protocol was developed prior to undertaking the review. To identify relevant studies on the review topic, we searched relevant databases, which cover the majority of the relevant literature, without language restrictions. We regard it therefore unlikely that we missed any relevant literature to the review topic. Furthermore, the review processes were rigorously implemented, with independent assessments taken at each stage, including literature screening and data extraction.

Limitations include the lack of studies on high fiber intake and iron status, and the limited number of studies on bowel function and growth in healthy children. This and the heterogeneity of studies prevented us from performing meta-analyses or subgroup analyses. Both RCTs had limited number of children included, which compromises statistical power. In addition, they were short-term studies which reduces their value for conclusions on growth. The studies based on the Generation R cohort (25, 26) only had a single dietary assessment at 12.9 or 14 months, respectively, and did not assess dietary changes over time. Calculation of dietary fiber intake using a FFQ can also be questioned. These studies also did not include separate analyses for children with high fiber intake.

## Conclusions

We found no putative association between moderately high fiber intake in infants and children up to 5 years from affluent countries and growth. If any potential effect exists, we consider such at best inconclusively limited. There is a lack of studies on high fiber intake and iron status in well-nourished infants and children. More studies are needed to clarify and elaborate on these observations. While positive effects of added dietary fiber on bowel function was shown in two short-term RCTs, this effect was not seen in the one observational cohort study included. More studies are warranted both to determine desirable levels of fiber intake in small children and to clarify the role of dietary fiber in infancy and childhood for normal bowel function, growth and nutritional status.

## Registration

Prospero registration number CRD42021288211.
