# Supplementary material for: Dietary fiber and growth, iron status and bowel function in children 0–5 years old: a systematic review
Source: Food Nutr Res. 2023 Mar 27;67:10.29219/fnr.v67.9011. doi: 10.29219/fnr.v67.9011 (PMC10084507; doi:10.29219/fnr.v67.9011)
Supplement: Supplementary file 1 [file FNR-67-9011-s001.docx]

Supplemental Table 1: Search strategy

All searches were performed on 15th. Nov .2021 by academic librarian Sabina Gillsund and Narcisa Hannerz at the Karolinska Institutet University library. Search strategies were peer reviewd by Hilde Strømme, University of Oslo Library of Medicine and Science.

Documentation of search strategies

University Library search consultation group

Date: **November 2021**

Topic/research question: **High dietary fiber intake in children and growth, bowel function and iron status**

Name of researcher(s): Agneta Åberg & Jutta Dierkes

Librarian(s): Narcisa Hannerz & Sabina Gillsund

Databases:

1. Medline (Ovid)
2. Embase (embase.com)
3. Cochrane (Wiley)
4. Scopus (Elsevier)

Total number of hits:

- Before deduplication: 9,414
- After deduplication: 5,643

Comments:

1. Medline

| Interface: Ovid MEDLINE(R) and Epub Ahead of Print, In-Process & Other Non-Indexed Citations and Daily  Date of Search: 15^th^ November 2021  Number of hits: 2,265  Comment: In Ovid, two or more words are automatically searched as phrases; i.e. no quotation marks are needed | Field labels   - exp/ = exploded MeSH term - / = non exploded MeSH term - .ti,ab,kf. = title, abstract and author keywords - adjx = within x words, regardless of order - * = truncation of word for alternate endings |
| --- | --- |
| Database(s): **Ovid MEDLINE(R) and Epub Ahead of Print, In-Process, In-Data-Review & Other Non-Indexed Citations and Daily**1946 to November 12, 2021 Search Strategy:   \| **#** \| **Searches** \| **Results** \| \| --- \| --- \| --- \| \| 1 \| Dietary Fiber/ \| 18336 \| \| 2 \| Polysaccharides/ \| 46584 \| \| 3 \| ((fiber* or fibre* or roughage* or wheat bran*) and (ate or bean? or cereal* or consum* or diet* or eat or eating or fed or feed or food or foods or fruit? or grain? or intake* or ingest* or legume? or nutrient* or nutrition or pea or peas or plant* or pulse? or vegan or vegetable? or vegetarian)).ti,ab,kf. \| 66113 \| \| 4 \| (glycan? or polysaccharide?).ti,ab,kf. \| 97223 \| \| 5 \| or/1-4 \| 182835 \| \| 6 \| Child, Preschool/ \| 961076 \| \| 7 \| Infant/ \| 830823 \| \| 8 \| (baby or babies or child* or infant? or infancy or toddler?).ti,ab,kf. \| 1879370 \| \| 9 \| (("1" or "2" or "3" or "4" or "5") adj3 year? adj3 (age* or old*)).ti,ab,kf. \| 237511 \| \| 10 \| (("6" or "7" or "8" or "9" or "10" or "11" or "12" or "13" or "14" or "15" or "16" or "17" or "18" or "19" or "20" or "21" or "22" or "23" or "24") adj3 month? adj3 (age* or old*)).ti,ab,kf. \| 126288 \| \| 11 \| or/6-10 \| 2512714 \| \| 12 \| exp Body Composition/ \| 59678 \| \| 13 \| exp Body Size/ \| 525246 \| \| 14 \| exp "Body Weights and Measures"/ \| 650769 \| \| 15 \| Growth/ \| 26450 \| \| 16 \| (growth or height or weight or overweight or obesity or obese or "body mass index" or bmi).ti,ab,kf. \| 2792450 \| \| 17 \| ((body or mass) adj2 (composition or fat or fat-free)).ti,ab,kf. \| 83848 \| \| 18 \| or/12-17 \| 3047710 \| \| 19 \| exp Anemia, Iron-Deficiency/ \| 10865 \| \| 20 \| Erythrocyte Indices/ \| 5879 \| \| 21 \| Ferritins/ \| 20434 \| \| 22 \| Hemoglobins/ \| 70669 \| \| 23 \| Hematocrit/ \| 33573 \| \| 24 \| Hepcidins/ \| 3342 \| \| 25 \| Iron/ \| 99457 \| \| 26 \| Iron, Dietary/ \| 3031 \| \| 27 \| Receptors, Transferrin/ \| 6632 \| \| 28 \| Transferrin/ \| 17219 \| \| 29 \| (anemi* or anaemi* or ferritin* or haemoglobin* or hematocrit or hemoglobin* or hepcidin* or isoferritin* or nonanaemi* or nonanemi* or serotransferrin* or siderophilin* or transferrin or transferrins).ti,ab,kf. \| 379004 \| \| 30 \| ((fe or iron) adj3 (deficien* or diet* or indices* or intake* or level* or micronutrient* or nutrient* or nutriti* or saturation* or source* or status or store* or supplement*)).ti,ab,kf. \| 50016 \| \| 31 \| or/19-30 \| 502567 \| \| 32 \| Abdominal Pain/ \| 22433 \| \| 33 \| Constipation/ \| 14887 \| \| 34 \| Defecation/ \| 7413 \| \| 35 \| exp Diarrhea/ \| 55013 \| \| 36 \| Digestion/ \| 23435 \| \| 37 \| Dysbiosis/ \| 4886 \| \| 38 \| Encopresis/ \| 666 \| \| 39 \| exp Gastrointestinal Absorption/ \| 45175 \| \| 40 \| exp Gastrointestinal Motility/ \| 37891 \| \| 41 \| Irritable Bowel Syndrome/ \| 8195 \| \| 42 \| (colicky pain or colonic inertia or colonospasm or constipation or defecation* or defaecation or duodenum acid* or duodenum pH or diarrhea or diarrhoea or digestion or dysbacterios* or dysbios* or dyschezia or dyspep* or encopresis or epigastric or fat resorption or functional colonic disease* or GI transit or ileum contraction or intestin* adaptation or intestin* reabsorption or intestine* tone or malabsorp* or obstipation or rect* motility or stool habit*).ti,ab,kf. \| 285092 \| \| 43 \| ((abdomen or abdominal or bowel or colon* or duoden* or gastr* or gut or ileal or ileum or intestin* or stomach) adj2 (absorption* or contract* or dysmotility or emptying or function* or habit* or motilities or motility or motion or movement* or pain or peristalsis or permeability or propulsion or transit)).ti,ab,kf. \| 156003 \| \| 44 \| ((fat or glucose or iron or jejunal or jejunum or lipid* or rectal or rectum) adj2 (absorbenc* or absorption*)).ti,ab,kf. \| 11422 \| \| 45 \| ((irritable or mucomembranous or mucous or spastic or spasm or unstable) adj2 (bowel or colon or coliti*)).ti,ab,kf. \| 15455 \| \| 46 \| or/32-45 \| 507198 \| \| 47 \| 18 or 31 or 46 \| 3888472 \| \| 48 \| 5 and 11 and 47 \| 2590 \| \| 49 \| 5 and 11 and 32 \| 44 \| \| 50 \| 5 and 11 and 46 \| 830 \| \| 51 \| 48 not (animals not humans).sh. \| 2275 \| \| 52 \| limit 51 to (congress or consensus development conference or consensus development conference, nih or editorial or festschrift or guideline or historical article or interview or lecture or letter or "meta analysis" or news or newspaper article or personal narrative or portrait or "review" or "systematic review") \| 460 \| \| 53 \| 51 not 52 \| 1815 \| \| 54 \| 51 not (editorial or interview or letter or comment or congress or legal case or meeting abstract).pt. \| 2265 \| | |

2. Embase

| Interface: embase.com  Date of Search: 15^th^ November 2021  Number of hits: 2,789  Comment: Emtree is the controlled vocabulary in Embase | Field labels   - /exp = exploded Emtree term - /de = non exploded Emtree term - ti,ab,kw = title, abstract and author keywords - NEAR/x = within x words, regardless of order - * = truncation of word for alternate endings |
| --- | --- |
| \| No. \| Query \| Results \| \| --- \| --- \| --- \| \| #1 \| 'dietary fiber'/de \| 23805 \| \| #2 \| 'fiber intake'/de \| 825 \| \| #3 \| 'high fiber diet'/de \| 2312 \| \| #4 \| 'low fiber diet'/de \| 249 \| \| #5 \| 'polysaccharide'/de \| 50662 \| \| #6 \| (fiber*:ti,ab,kw OR fibre*:ti,ab,kw OR roughage*:ti,ab,kw OR 'wheat bran*':ti,ab,kw) AND (ate:ti,ab,kw OR bean$:ti,ab,kw OR cereal*:ti,ab,kw OR consum*:ti,ab,kw OR diet*:ti,ab,kw OR eat:ti,ab,kw OR eating:ti,ab,kw OR fed:ti,ab,kw OR feed:ti,ab,kw OR food:ti,ab,kw OR foods:ti,ab,kw OR fruit$:ti,ab,kw OR grain$:ti,ab,kw OR intake*:ti,ab,kw OR ingest*:ti,ab,kw OR legume$:ti,ab,kw OR nutrient*:ti,ab,kw OR nutrition:ti,ab,kw OR pea:ti,ab,kw OR peas:ti,ab,kw OR plant$:ti,ab,kw OR pulse$:ti,ab,kw OR vegan:ti,ab,kw OR vegetable$:ti,ab,kw OR vegetarian:ti,ab,kw) \| 75883 \| \| #7 \| glycan$:ti,ab,kw OR polysaccharide$:ti,ab,kw \| 111334 \| \| #8 \| #1 OR #2 OR #3 OR #4 OR #5 OR #6 OR #7 \| 206101 \| \| #9 \| 'baby'/de \| 14154 \| \| #10 \| 'infant'/de \| 745404 \| \| #11 \| 'preschool child'/de \| 659331 \| \| #12 \| 'toddler'/de \| 5579 \| \| #13 \| baby:ti,ab,kw OR babies:ti,ab,kw OR child*:ti,ab,kw OR infant$:ti,ab,kw OR infancy:ti,ab,kw OR toddler$:ti,ab,kw \| 2414507 \| \| #14 \| (('1' OR '2' OR '3' OR '4' OR '5') NEAR/3 year$ NEAR/3 (age* OR old*)):ti,ab,kw \| 480560 \| \| #15 \| (('6' OR '7' OR '8' OR '9' OR '10' OR '11' OR '12' OR '13' OR '14' OR '15' OR '16' OR '17' OR '18' OR '19' OR '20' OR '21' OR '22' OR '23' OR '24') NEAR/3 month$ NEAR/3 (age* OR old*)):ti,ab,kw \| 164482 \| \| #16 \| #9 OR #10 OR #11 OR #12 OR #13 OR #14 OR #15 \| 3153328 \| \| #17 \| 'body composition'/exp \| 113466 \| \| #18 \| 'body height'/exp \| 78917 \| \| #19 \| 'body growth'/exp \| 124974 \| \| #20 \| 'body mass'/exp \| 511629 \| \| #21 \| 'body size'/exp \| 28127 \| \| #22 \| 'body weight'/exp \| 774637 \| \| #23 \| growth:ti,ab,kw OR height:ti,ab,kw OR weight:ti,ab,kw OR overweight:ti,ab,kw OR obesity:ti,ab,kw OR obese:ti,ab,kw OR 'body mass index':ti,ab,kw OR bmi:ti,ab,kw \| 3619224 \| \| #24 \| ((body OR mass) NEAR/2 (composition OR fat OR 'fat free')):ti,ab,kw \| 114402 \| \| #25 \| #17 OR #18 OR #19 OR #20 OR #21 OR #22 OR #23 OR #24 \| 3997155 \| \| #26 \| 'ferritin'/de \| 53929 \| \| #27 \| 'hematocrit'/de \| 73772 \| \| #28 \| 'hemoglobin'/de \| 218934 \| \| #29 \| 'hepcidin'/de \| 9013 \| \| #30 \| 'iron'/de \| 188524 \| \| #31 \| 'iron deficiency anemia'/exp \| 31960 \| \| #32 \| 'iron intake'/de \| 4994 \| \| #33 \| 'mean corpuscular volume'/de \| 16859 \| \| #34 \| 'transferrin'/de \| 33451 \| \| #35 \| 'transferrin receptor'/de \| 9623 \| \| #36 \| anemi*:ti,ab,kw OR anaemi*:ti,ab,kw OR ferritin*:ti,ab,kw OR haemoglobin*:ti,ab,kw OR hematocrit:ti,ab,kw OR hemoglobin*:ti,ab,kw OR hepcidin*:ti,ab,kw OR isoferritin*:ti,ab,kw OR nonanaemi*:ti,ab,kw OR nonanemi*:ti,ab,kw OR serotransferrin*:ti,ab,kw OR siderophilin*:ti,ab,kw OR transferrin:ti,ab,kw OR transferrins:ti,ab,kw \| 541799 \| \| #37 \| ((fe OR iron) NEAR/3 (deficien* OR diet* OR indices* OR intake* OR level* OR micronutrient* OR nutrient* OR nutriti* OR saturation* OR source* OR status OR store* OR supplement*)):ti,ab,kw \| 70693 \| \| #38 \| #26 OR #27 OR #28 OR #29 OR #30 OR #31 OR #32 OR #33 OR #34 OR #35 OR #36 OR #37 \| 812380 \| \| #39 \| 'abdominal pain'/exp \| 188101 \| \| #40 \| 'intestine function'/exp \| 82955 \| \| #41 \| 'intestine function disorder'/exp \| 473118 \| \| #42 \| 'digestion'/de \| 33645 \| \| #43 \| 'defecation'/exp \| 15408 \| \| #44 \| 'defecation habit'/exp \| 4220 \| \| #45 \| 'feces incontinence'/exp \| 22682 \| \| #46 \| 'gastrointestinal absorption'/exp \| 143525 \| \| #47 \| 'gastrointestinal absorption'/de \| 4435 \| \| #48 \| 'gastrointestinal motility'/exp \| 35639 \| \| #49 \| 'irritable colon'/de \| 28736 \| \| #50 \| 'colicky pain':ti,ab,kw OR 'colonic inertia':ti,ab,kw OR colonospasm:ti,ab,kw OR constipation:ti,ab,kw OR defecation*:ti,ab,kw OR defaecation:ti,ab,kw OR 'duodenum acid*':ti,ab,kw OR 'duodenum ph':ti,ab,kw OR diarrhea:ti,ab,kw OR diarrhoea:ti,ab,kw OR digestion:ti,ab,kw OR dysbacteriosis:ti,ab,kw OR dysbiosis:ti,ab,kw OR dyschezia:ti,ab,kw OR dyspep*:ti,ab,kw OR encopresis:ti,ab,kw OR epigastric:ti,ab,kw OR 'fat resorption':ti,ab,kw OR 'functional colonic disease*':ti,ab,kw OR 'gi transit':ti,ab,kw OR 'ileum contraction':ti,ab,kw OR 'intestin* adaptation':ti,ab,kw OR 'intestin* reabsorption':ti,ab,kw OR 'intestine* tone':ti,ab,kw OR malabsorp*:ti,ab,kw OR obstipation:ti,ab,kw OR 'rect* motility':ti,ab,kw OR 'stool habit*':ti,ab,kw \| 397486 \| \| #51 \| ((abdomen OR abdominal OR bowel OR colon OR duoden* OR gastr* OR gut OR intestin* OR stomach) NEAR/2 (absorption* OR contract* OR dysmotilities OR dysmotility OR function* OR habit* OR motilities OR motility OR motion OR movement* OR pain OR transit)):ti,ab,kw \| 212043 \| \| #52 \| ((fat OR glucose OR iron OR jejunal OR jejunum OR lipid* OR rectal OR rectum) NEAR/2 (absorbenc* OR absorption*)):ti,ab,kw \| 14973 \| \| #53 \| ((irritable OR mucomembranous OR mucous OR spastic OR spasm OR unstable) NEAR/2 (bowel OR colon OR coliti*)):ti,ab,kw \| 24867 \| \| #54 \| #39 OR #40 OR #41 OR #42 OR #43 OR #44 OR #45 OR #46 OR #47 OR #48 OR #49 OR #50 OR #51 OR #52 OR #53 \| 1054812 \| \| #55 \| #25 OR #38 OR #54 \| 5495962 \| \| #56 \| #8 AND #16 AND #55 \| 4298 \| \| #57 \| #56 NOT ([animals]/lim NOT [humans]/lim) \| 3805 \| \| #58 \| #57 AND ('conference abstract'/it OR 'conference paper'/it OR 'conference review'/it OR 'editorial'/it OR 'letter'/it OR 'note'/it OR 'short survey'/it) \| 1016 \| \| #59 \| #57 NOT #58 \| 2789 \| | |

3. Cochrane Library

| Interface: Wiley  Date of Search: 15^th^ November 2021  Number of hits: 790 | Field labels   - ti,ab,kw = title, abstract and author keywords - NEAR/x = within x words, regardless of order - * = truncation of word for alternate endings |
| --- | --- |
| \| #1 \| MeSH descriptor: [Dietary Fiber] this term only \| 1812 \| \| --- \| --- \| --- \| \| #2 \| MeSH descriptor: [Polysaccharides] this term only \| 609 \| \| #3 \| ((fiber* OR fibre* OR roughage* OR (wheat NEXT bran*)) AND (ate OR bean OR beans OR cereal* OR consum* OR diet* OR eat OR eating OR fed OR feed OR food OR foods OR fruit OR fruits OR grain OR grains OR intake* OR ingest* OR legume OR legumes OR nutrient* OR nutrition OR pea OR peas OR plant* OR pulse OR pulses OR vegan OR vegetable OR vegetables OR vegetarian)):ti,ab,kw \| 7388 \| \| #4 \| (glycan OR glycans OR polysaccharide OR polysaccharides):ti,ab,kw \| 2984 \| \| #5 \| #1 OR #2 OR #3 OR #4 \| 10170 \| \| #6 \| MeSH descriptor: [Child, Preschool] this term only \| 30637 \| \| #7 \| MeSH descriptor: [Infant] this term only \| 22740 \| \| #8 \| (baby or babies or child* or infant or infants or infancy or toddler or toddlers):ti,ab,kw \| 202864 \| \| #9 \| (("1" or "2" or "3" or "4" or "5") NEAR/3 year* NEAR/3 (age* or old*)):ti,ab,kw \| 77139 \| \| #10 \| (("6" or "7" or "8" or "9" or "10" or "11" or "12" or "13" or "14" or "15" or "16" or "17" or "18" or "19" or "20" or "21" or "22" or "23" or "24") NEAR/3 month* NEAR/3 (age* or old*)):ti,ab,kw \| 16068 \| \| #11 \| #6 OR #7 OR #8 OR #9 OR #10 \| 253668 \| \| #12 \| MeSH descriptor: [Body Composition] explode all trees \| 5484 \| \| #13 \| MeSH descriptor: [Body Size] explode all trees \| 30735 \| \| #14 \| MeSH descriptor: [Body Weights and Measures] explode all trees \| 34050 \| \| #15 \| MeSH descriptor: [Growth] explode all trees \| 21026 \| \| #16 \| (growth or height or weight or overweight or obesity or obese or "body mass index" or bmi):ti,ab,kw \| 221146 \| \| #17 \| ((body or mass) NEAR/2 (composition or fat or "fat-free")):ti,ab,kw \| 21228 \| \| #18 \| #12 OR #13 OR #14 OR #15 OR #16 OR #17 \| 228005 \| \| #19 \| MeSH descriptor: [Anemia, Iron-Deficiency] this term only \| 1405 \| \| #20 \| MeSH descriptor: [Erythrocyte Indices] this term only \| 176 \| \| #21 \| MeSH descriptor: [Ferritins] this term only \| 1049 \| \| #22 \| MeSH descriptor: [Hemoglobins] this term only \| 3352 \| \| #23 \| MeSH descriptor: [Hematocrit] this term only \| 1561 \| \| #24 \| MeSH descriptor: [Hepcidins] this term only \| 152 \| \| #25 \| MeSH descriptor: [Iron] this term only \| 2600 \| \| #26 \| MeSH descriptor: [Iron, Dietary] this term only \| 415 \| \| #27 \| MeSH descriptor: [Receptors, Transferrin] this term only \| 123 \| \| #28 \| MeSH descriptor: [Transferrin] this term only \| 374 \| \| #29 \| (anemi* OR anaemi* OR ferritin* OR haemoglobin* OR hematocrit OR hemoglobin* OR hepcidin* OR isoferritin* OR nonanaemi* OR nonanemi* OR serotransferrin* OR siderophilin* OR transferrin OR transferrins):ti,ab,kw \| 61012 \| \| #30 \| ((fe OR iron) NEAR/3 (deficien* OR diet* OR indices* OR intake* OR level* OR micronutrient* OR nutrient* OR nutriti* OR saturation* OR source* OR status OR store* OR supplement*)):ti,ab,kw \| 6981 \| \| #31 \| #19 OR #20 OR #21 OR #22 OR #23 OR #24 OR #25 OR #26 OR #27 OR #28 OR #29 OR #30 \| 62747 \| \| #32 \| MeSH descriptor: [Abdominal Pain] this term only \| 1126 \| \| #33 \| MeSH descriptor: [Constipation] this term only \| 1827 \| \| #34 \| MeSH descriptor: [Defecation] this term only \| 854 \| \| #35 \| MeSH descriptor: [Diarrhea] explode all trees \| 3640 \| \| #36 \| MeSH descriptor: [Digestion] this term only \| 338 \| \| #37 \| MeSH descriptor: [Dysbiosis] this term only \| 98 \| \| #38 \| MeSH descriptor: [Encopresis] this term only \| 34 \| \| #39 \| MeSH descriptor: [Gastrointestinal Absorption] explode all trees \| 2171 \| \| #40 \| MeSH descriptor: [Gastrointestinal Motility] explode all trees \| 3004 \| \| #41 \| MeSH descriptor: [Irritable Bowel Syndrome] this term only \| 1289 \| \| #42 \| ("colicky pain" OR "colonic inertia" OR colonospasm OR constipation OR defecation* OR defaecation OR (duodenum NEXT acid*) OR "duodenum pH" OR diarrhea OR diarrhoea OR digestion OR dysbacteriosis OR dysbiosis OR dyschezia OR dyspep* OR encopresis OR epigastric OR "fat resorption" OR ("functional colonic" NEXT disease*) OR "GI transit" OR "ileum contraction" OR (intestin* NEXT adaptation) OR (intestin* NEXT reabsorption) OR (intestine* NEXT tone) OR malabsorp* OR obstipation OR (rect* NEXT motility) OR (stool NEXT habit*)):ti,ab,kw \| 49546 \| \| #43 \| ((abdomen OR abdominal OR bowel OR colon OR duoden* OR gastr* OR gut OR ileal OR ileum OR intestin* OR stomach) NEAR/2 (absorption* OR contract* OR dysmotilities OR dysmotility OR emptying OR function* OR habit* OR motilities OR motility OR motion OR movement* OR pain OR peristalsis OR permeability OR propulsion OR transit)):ti,ab,kw \| 31473 \| \| #44 \| ((fat OR glucose OR iron OR jejunal OR jejunum OR lipid* OR rectal OR rectum) NEAR/2 (absorbence* OR absorption*)):ti,ab,kw \| 1620 \| \| #45 \| ((irritable OR mucomembranous OR mucous OR spastic OR spasm OR unstable) NEAR/2 (bowel OR colon OR coliti*)):ti,ab,kw \| 4236 \| \| #46 \| #32 OR #33 OR #34 OR #35 OR #36 OR #37 OR #38 OR #39 OR #40 OR #41 OR #42 OR #43 OR #44 OR #45 \| 71340 \| \| #47 \| #18 OR #31 OR #46 \| 324574 \| \| #48 \| #5 AND #11 AND #47 \| 809 \| \| #49 \| #48 in Trials \| 790 \| | |

4. Scopus

| Interface: Elsevier  Date of Search: 15^th^ November 2021  Number of hits: 3,570 | Field labels   - TITLE-ABS-KEY = title, abstract and author keywords - W/x = within x words, regardless of order - * = truncation of word for alternate endings |
| --- | --- |
| ( ( TITLE-ABS-KEY ( ( ( baby OR babies OR child* OR infant# OR infancy OR toddler# ) OR ( ( ( "1" OR "2" OR "3" OR "4" OR "5" ) W/2 year# ) W/2 ( age* OR old* ) ) OR ( ( ( "4" OR "5" OR "6" OR "7" OR "8" OR "9" OR "10" OR "11" OR "12" OR "13" OR "14" OR "15" OR "16" OR "17" OR "18" OR "19" OR "20" OR "21" OR "22" OR "23" OR "24" ) W/2 month# ) W/2 ( age* OR old* ) ) ) ) ) ) AND ( ( TITLE-ABS-KEY ( ( fiber* OR fibre* OR roughage* OR "wheat bran*" ) AND ( ate OR bean# OR cereal* OR consum* OR diet* OR eat# OR fed OR feed OR food OR foods OR fruit# OR grain# OR intake* OR ingest* OR legume# OR nutrient* OR nutrition OR pea OR peas OR plant* OR pulse# OR vegan OR vegetable# OR vegetarian ) ) ) ) AND ( ( TITLE-ABS-KEY ( growth OR height OR weight OR overweight OR obesity OR obese OR "body mass index" OR bmi ) ) OR ( TITLE-ABS-KEY ( ( body OR mass ) W/1 ( composition OR fat OR "fat-free" ) ) ) OR ( TITLE-ABS-KEY ( anemi* OR anaemi* OR ferritin* OR haemoglobin* OR hematocrit OR hemoglobin* OR hepcidin* OR isoferritin* OR nonanaemi* OR nonanemi* OR serotransferrin* OR siderophilin* OR transferrin OR transferrins ) ) OR ( TITLE-ABS-KEY ( ( fe OR iron ) W/2 ( deficien* OR diet* OR indices* OR intake* OR level* OR micronutrient* OR nutrient* OR nutriti* OR saturation* OR source* OR status OR store* OR supplement* ) ) ) OR ( ( TITLE-ABS-KEY ( "colicky pain" OR "colonic inertia" OR colonospasm OR constipation OR defecation* OR defaecation OR "duodenum acid*" OR "duodenum pH" OR diarrhea OR diarrhoea OR digestion OR dysbacteriosis OR dysbiosis OR dyschezia OR dyspep* OR encopresis OR epigastric OR "fat resorption" OR "functional colonic disease*" OR "GI transit" OR "ileum contraction" OR "intestin* adaptation" OR "intestin* reabsorption" OR "intestine* tone" OR malabsorp* OR obstipation OR "rect* motility" OR "stool habit*" ) ) ) OR ( TITLE-ABS-KEY ( ( abdomen OR abdominal OR bowel OR colon* OR duoden* OR gastr* OR gut OR ileal OR ileum OR intestin* OR stomach ) W/2 ( absorption* OR contract* OR dysmotilities OR dysmotility OR emptying OR function* OR habit* OR motilities OR motility OR motion OR movement* OR pain OR peristalsis OR permeability OR propulsion OR transit ) ) ) OR ( TITLE-ABS-KEY ( ( fat OR glucose OR iron OR jejunal OR jejunum OR lipid* OR rectal OR rectum ) W/2 ( absorbenc* OR absorption* ) ) ) OR ( TITLE-ABS-KEY ( ( irritable OR mucomembranous OR mucous OR spastic OR spasm OR unstable ) W/1 ( bowel OR colon OR coliti* ) ) ) ) AND ( EXCLUDE ( DOCTYPE , "re" ) OR EXCLUDE ( DOCTYPE , "cp" ) OR EXCLUDE ( DOCTYPE , "ch" ) OR EXCLUDE ( DOCTYPE , "le" ) OR EXCLUDE ( DOCTYPE , "no" ) OR EXCLUDE ( DOCTYPE , "ed" ) OR EXCLUDE ( DOCTYPE , "sh" ) OR EXCLUDE ( DOCTYPE , "cr" ) OR EXCLUDE ( DOCTYPE , "bk" ) ) | |

Supplementary Table 2: Excluded studies after fulltext screening

| **Author, Title** | **Year** | **Reason for exclusion** |
| --- | --- | --- |
| Alanís, S.E., J. Meijerink, and L. Vega-Franco, Dietary fiber in the medical treatment in various pediatric problems. Boletin medico del Hospital Infantil de Mexico, 1983. 40(2): p. 67-71. | 1983 | Treatment. Review. Spanish |
| Basturk, A., R. Artan, and A. Yilmaz, Efficacy of synbiotic, probiotic, and prebiotic treatments for irritable bowel syndrome in children: a randomized controlled trial. Journal of pediatric gastroenterology and nutrition, 2016. 62: p. 421‐. | 2016 | Treatment for IBS. Inulin or probiotic supplements. |
| Becker, M. and R. Rosskamp, Therapy of constipation with wheat bran in infancy and early childhood. Therapie der Obstipation mit Weizenkleie im Sauglings- und Kleinkindesalter., 1987. 135(8): p. 522-524. | 1987 | Treatment for constipation (but does look at iron status too) |
| Buyken, A.E., et al., Relation of dietary glycemic index, glycemic load, added sugar intake, or fiber intake to the development of body composition between ages 2 and 7 y. The American journal of clinical nutrition, 2008. 88(3): p. 755-762. | 2008 | Only BMI and body fat % |
| Castillejo, G., et al., A controlled, randomized, double-blind trial to evaluate the effect of a supplement of cocoa husk that is rich in dietary fiber on colonic transit in constipated pediatric patients. Pediatrics, 2006. 118(3): p. e641-648. | 2006 | Treatment of constipation. Cocoa Husk supplement. |
| Chao, H.C., et al., Cutoff volume of dietary fiber to ameliorate constipation in children. The Journal of pediatrics, 2008. 153(1): p. 45-49. | 2008 | Treatment of constipation. |
| Davidsson, L., et al., Dietary fiber in weaning cereals: a study of the effect on stool characteristics and absorption of energy, nitrogen, and minerals in healthy infants. Journal of pediatric gastroenterology and nutrition, 1996. 22(2): p. 167-179. | 1996 | <6 months |
| García, C.I.O., B.G. de Luna, and O.T. Reynoso, Dietary and laxative management in pediatric patients with chronic functional constipation without fecal incontinency. Acta Gastroenterologica Latinoamericana, 2017. 47(1): p. 8-13. | 2017 | Treatment of constipation. |
| Inoue, R., et al., Dietary supplementation with partially hydrolyzed guar gum helps improve constipation and gut dysbiosis symptoms and behavioral irritability in children with autism spectrum disorder. Journal of clinical biochemistry and nutrition, 2019. 64(3): p. 217-223. | 2019 | Treatment of constipation, guar gum supplement. |
| Kadim, M., et al., Effect of Supplementation of Zinc, Glutamine, Fiber, and Prebiotics in Presumed Healthy Indonesian Children Aged 1-3 Years. Pediatric gastroenterology, hepatology & nutrition, 2020. 23(4): p. 388-396. | 2020 | Combined Zinc, glutamine, fiber and prebiotics supplement |
| Kokke, F.T.M., et al., A dietary fiber mixture versus lactulose in the treatment of childhood constipation: a double-blind randomized controlled trial. Journal of pediatric gastroenterology and nutrition, 2008. 47(5): p. 592-597. | 2008 | Treatment of constipation. |
| Larrosa, S., et al., Fibre Intake Is Associated with Cardiovascular Health in European Children. Nutrients, 2020. 13(1). | 2020 | Only cross-sectional analysis of fiber intake and BMI at 8 years |
| Loening-Baucke, V., E. Miele, and A. Staiano, Fiber (glucomannan) is beneficial in the treatment of childhood constipation. Pediatrics, 2004. 113(3): p. e259-264. | 2004 | Treatment of constipation, glucomannan |
| Lungu, E., et al., Higher Fiber Complementary Food Alters Fecal Microbiota Composition And Normalizes Stool Form In Malawian Children: A Randomized Trial. African Journal of Food, Agriculture, Nutrition and Development, 2021. 21(4): p. 17854-17875. | 2021 | Malawi, undernourished |
| Maffei, H.V.L. and A.P. Vicentini, Prospective evaluation of dietary treatment in childhood constipation: high dietary fiber and wheat bran intake are associated with constipation amelioration. Journal of pediatric gastroenterology and nutrition, 2011. 52(1): p. 55-59. | 2011 | Treatment of constipation. |
| Mooren, G., et al., The connection between dietary fibre intake and chronic constipation in children. Nederlands tijdschrift voor geneeskunde., 1996. 140(41): p. 2036‐2039. | 1996 | Treatment of constipation |
| Nguyen, A.N., et al., Carbohydrate Intake in Early Childhood and Body Composition and Metabolic Health: Results from the Generation R Study. Nutrients, 2020. 12(7). | 2020 | Fiber intake not reported |
| Okubo, H., et al., Rate of eating in early life is positively associated with current and later body mass index among young Japanese children: the Osaka Maternal and Child Health Study. Nutrition research (New York, N.Y.), 2017. 37: p. 20-28. | 2017 | Fiber intake not reported |
| Payne, J.A. and N.R. Belton, Nutrient intake and growth in preschool children. I. Comparison of energy intake and sources of energy with growth. Journal of Human Nutrition and Dietetics, 1992. 5(5): p. 287-298. | 1992 | Associations between fibre and anthropometry not reported |
| Pospisil, C., A.F. Czernitzki, and C. Scheffler, No association between nutrition and body height in German kindergarten children - a pilot study. Anthropologischer Anzeiger; Bericht uber die biologisch-anthropologische Literatur, 2017. 74(3): p. 199-202. | 2017 | Cross-sectional |
| Quitadamo, P., et al., A randomized, prospective, comparison study of a mixture of acacia fiber, psyllium fiber, and fructose vs polyethylene glycol 3350 with electrolytes for the treatment of chronic functional constipation in childhood. The Journal of pediatrics, 2012. 161(4): p. 710-715.e711. | 2012 | Treatment of constipation, acacia and psyllium fiber |
| Rose, C.M., L.L. Birch, and J.S. Savage, Dietary patterns in infancy are associated with child diet and weight outcomes at 6 years. International journal of obesity (2005), 2017. 41(5): p. 783-788. | 2017 | Fiber intake not analyzed |
| Sullivan, P.B., et al., Effectiveness of using a behavioural intervention to improve dietary fibre intakes in children with constipation. Journal of human nutrition and dietetics : the official journal of the British Dietetic Association, 2012. 25(1): p. 33-42. | 2012 | Treatment of constipation. |
| Toporovski, M.S., et al., Effect of Polydextrose/Fructooligosaccharide Mixture on Constipation Symptoms in Children Aged 4 to 8 Years. Nutrients, 2021. 13(5). | 2021 | Treatment of constipation. |
| Treem, W.R., et al., Evaluation of the effect of a fiber-enriched formula on infant colic. The Journal of pediatrics, 1991. 119(5): p. 695-701. | 1991 | Treatment of colic. Polysaccharide supplement. |
| Ustundag, G., et al., Can partially hydrolyzed guar gum be an alternative to lactulose in treatment of childhood constipation? The Turkish journal of gastroenterology : the official journal of Turkish Society of Gastroenterology, 2010. 21(4): p. 360-364. | 2010 | Treatment of constipation, guar gum supplement. |
| Weber, T.K., et al., Dietary fiber mixture in pediatric patients with controlled chronic constipation. Journal of pediatric gastroenterology and nutrition, 2014. 58(3): p. 297-302. | 2014 | Treatment of constipation, fiber/prebiotics supplement |
| Zoppi, G., et al., Potential complications in the use of wheat bran for constipation in infancy. Journal of pediatric gastroenterology and nutrition, 1982. 1(1): p. 91-95. | 1982 | Treatment of constipation. No control group. |
